# In Silico–Ex Vitro Iteration Strategy for Affinity Maturation of Anti-Ricin Peptides and the SPR Biosensing Application

**DOI:** 10.3390/toxins15080490

**Published:** 2023-08-03

**Authors:** Zhifang Yang, Chuang Wang, Jia Liu, Lan Xiao, Lei Guo, Jianwei Xie

**Affiliations:** 1 State Key Laboratory of Toxicology and Medical Countermeasures, and Laboratory of Toxicant Analysis, Institute of Pharmacology and Toxicology, Academy of Military Medical Sciences, Beijing 100850, China; 2 Key Laboratory of Ethnomedicine Ministry of Education (Minzu University of China), School of Pharmacy, Minzu University of China, Beijing 100081, China; 3 College of Pharmacy, Hebei Science and Technology University, Shijiazhuang 050018, China

**Keywords:** affinity maturation, phage display, ricin, peptide, molecular docking, biosensing, surface plasmon resonance

## Abstract

The highly toxic plant toxin ricin is one of the most known threatening toxins. Accurate and sensitive biosensing methods for the first emergency response and intoxication treatment, are always pursued in the biodefense field. Screening affinity molecules is the fundamental mainstream approach for developing biosensing methods. Compared with common affinity molecules such as antibodies and oligonucleotide aptamers, peptides have great potential as biosensing modules with more accessible chemical synthesis capability and better batch-to-batch stability than antibodies, more abundant interaction sites, and robust sensing performance towards complex environments. However, anti-ricin peptides are so scant to be screened and discovered, and an advanced screening strategy is the utmost to tackle this issue. Here, we present a new in silico-in vitro iteration-assisted affinity maturation strategy of anti-ricin peptides. We first obtained affinity peptides targeting ricin through phage display with five panning rounds of “coating-elution-amplification-enrichment” procedures. The binding affinity and kinetic parameters characterized by surface plasmon resonance (SPR) showed that we had obtained four peptides owning dissociation constants (K_D_) around 2~35 μM, in which peptide PD-2-R5 has the lower K_D_ of 4.7 μM and higher stable posture to interact with ricin. We then constructed a new strategy for affinity maturity, composing two rounds of in silico-in vitro iterations. Firstly, towards the single-site alanine scanning mutation peptide library, the molecular docking predictions match the SPR evaluation results well, laying a solid foundation for designing a full saturation mutated peptide library. Secondly, plenty of in silico saturation mutation prediction results guided the discovery of peptides PD2-R5-T3 and PD-2-R5-T4 with higher affinity from only a limited number of SPR evaluation experiments. Both evolved peptides had increased affinity by about 5~20 times, i.e., K_D_ of 230 nM and 900 nM. A primary cellular toxicity assay indicated that both peptides could protect cells against ricin damage. We further established an SPR assay based on PD-2-R5-T3 and PD-2-R5-T4 elongated with an antifouling peptide linkage and achieved good linearity with a sensitivity of 1 nM and 0.5 nM, respectively. We hope this new affinity-mature strategy will find its favorable position in relevant peptide evolution, biosensing, and medical countermeasures for biotoxins to protect society’s security and human life better.

## 1. Introduction

Plant-derived protein toxins are secondary metabolites generated by the plants to protect themselves, including ribosome-inactivating proteins (RIPs), lectins, protease inhibitors, α-amylase inhibitors, canatoxin-like proteins and ureases, arcelins, antimicrobial peptides, pore-forming toxins, etc. [1]. These protein toxins may cause symptoms that include but are not exclusive to vomiting, nausea, psychological disorders, embryonic malformations, and cardiac dysrhythmias. In particular, RIPs are a class of protein toxins that act specifically on mRNA and inhibit ribosomal function [2]. According to the structural characteristics, RIPs can be divided into three types [3], in which the type II ribosome-inactivating protein (RIP-II) is a kind of biotoxin with N-glycosidase activity and widely exists in plants. The toxic mechanism of RIP-II is to act on specific nucleotide regions of the 28S ribosomal RNA α-Sarcin ricin ring (SRL) domain. The biotoxin can remove specific adenine (such as the adenine N-glycosidic bond at the A_4324_ site of 28S rRNA hydrolysis in eukaryotic cells), leading to ribosome inactivation, the protein synthesis is irreversibly inhibited, which eventually leads to cell death [2,4].

Ricin is the most famous RIP-II, which is so representative that it is the only biotoxin listed in both the International Convention for the Prohibition of Chemical Weapons and the International Convention for the Prohibition of Biological and Toxin Weapons [3,5]. The median lethal dose (LD_50_) of ricin for mice is 3~5 µg/kg by intravenous injection, and for humans is 1~20 mg/kg, equivalent to 3~12 castor beans [6]. No specific antidotes have been available in clinical applications till now. The most representative threat was the Markov ricin umbrella assassination in 1978, and several international white powder threats occurred frequently since 2000 [7].

In emergency response and intoxication treatment, developing rapid, sensitive, and accurate detection modules and sensing approaches for ricin is crucial. Most of the current biosensing methods are affinity-based, such as enzyme-linked immunosorbent assays (ELISA), fluorescent immunosorbent assays, the chemiluminescent immunoassay, and colloidal gold immunochromatographic assays on ricin using monoclonal (mAb) or polyclonal antibodies as recognition modules. In our previous work, we accurately distinguished and quantified ricin by mAb sandwiched surface plasmon resonance (SPR) with a concentration lowered to 0.6 ng/mL in different complex matrices, such as stevia, protein powder, and human plasma [8]. Additionally, we developed a double amplification strategy based on immunoGNPs with a limit of detection (LOD) of 0.3 ng/mL, in which the immunoGNPs provided enhanced signal amplification via SPR and nanozyme catalytic enhancement [9]. Similarly, oligonucleotide aptamers have also been employed as sensing modules in recent years. For example, P. Labuza’s group constructed an aptamer-based surface enhanced Raman scattering method to detect ricin within 40 min, in which the LOD was 10 ng/mL in phosphate-buffered saline, 50 ng/mL in orange juice, and 100 ng/mL in milk [10]. Sreevatsan’s group selected and characterized a single DNA aptamer SSRA1 against the ricin B chain and developed an SSRA1-based biosensor with a LOD of 30 ng/mL in liquid food matrices [11].

Besides antibodies and aptamers, a kind of affinity module, peptides recall attention due to the rapid progress of the peptide synthesis industry. Peptides have simpler structures than antibodies but keep more interaction sites than aptamers, and they have proper, good stability both in vitro and in vivo; no immunogenicity can be easily chemically modified, and they can be stored long term [12,13]. In the past decade, peptides have become the star molecules in the biosensing field.

Specific peptides toward their targets can be readily generated from various in vitro library screening techniques. Many screening approaches have been developed, such as phage display, yeast surface display, mRNA display, ribosome display, and covalent DNA display techniques [14]. Phage display is the current most widely used technique due to the advantages of large library capacity, simple operation, and low cost. After its establishment in 1990 by Smith’s group, phage display quickly proved its convenience and powerful efficiency towards different target molecules, such as cancer-specific peptides [15], derived human therapeutic antibodies [16,17], and covalent inhibitors [18]. In this technique, the foreign gene is inserted into the phage shell protein gene, and the foreign peptide or protein is expressed in the phage capsid protein [19,20]. Several bio-panning rounds were then performed to screen out the affinity peptides for target molecules.

So far, we overviewed the screened peptides of ricin biotoxin by phage display technique. In 2003, J Valdes’ group in the U.S. Army Edgewood Chemical and Biological Center screened the 12-mer peptide inhibitor P3 (WPHRHHHSEIGH) towards ricin through five rounds by the FliTrx random peptide combination library with the dissociation constant (K_D_) of 1 μM [21]. Similarly, J. Mantis’ group simulated the linear epitope of monoclonal antibodies (JB4, B/J F9, C/M A2) binding to the subdomain ricin B chain by Ph.D.-12^TM^ phage display peptide library and obtained C4 peptide (CLTSDSNIRETVVKI) with three rounds panning, which provided supporting information of the design vaccine epitope of a subdomain ricin B chain [22].

In general, depending on the nature of the phage itself, the peptides overexpressed on its surface exert good, even strong binding affinity to their targets. However, the inherent fundamental reason is multivalency, i.e., high avidity rather than high affinity. Multivalency is a good approach to creating ligands with higher avidity by combining multiple binding domains [23]. Still, the peptide itself might not be evolved to be the one with the highest affinity. Apart from other efforts from the view of enlarging the library capacity, such as mismatch PCR, site-directed mutation, or strand replacement during the process of phage display [24], here we try to seek the possibility of affinity maturation after phage display, which means, could we find a favorable way to further improve the affinity of a single peptide rather than multivalent composition? Therefore, we composed a residue variance-prone approach after conventional phage display procedures and achieved positive results.

In this work, we screened ricin-binding peptides using the Ph.D.-12^TM^ phage display peptide library. Toward several chosen affinity peptides, we created a novel affinity maturation strategy. The core concept of this strategy is the iteration uses of in silico molecular docking prediction and ex vitro SPR evaluation experimental results. By using this strategy, we achieved (i) a reliable concept of using virtual docking prediction to guide the experimental design so as to greatly save the consumed cost; (ii) two peptides with one order of magnitude affinity than parent peptide; a new affinity mutation strategy proved to be useful; (iii) potential inhibitors to ricin primarily proved by cellular toxicity assay; and (iv) a convenient SPR sensing assay with good quantification (0.5–100 nM) and sensitivity (0.5 or 1 nM). We hope this strategy and the evolved peptides have proven pivotal to the biothreat response, the medical countermeasures, and the forensic science.

## 2. Results and Discussion

The characteristics of simple operation and efficient output make phage display widely used for specific peptide or protein screening. However, the binding affinity values of most screened peptides were at the K_D_ of μM level. For example, Valdes’ group generated P3 peptide mimotopes against ricin through the *E. coli* FliTrx random peptide library in 2003, in which the K_D_ of P3 was 1 μM. The primary reason for the moderate affinity is that the phage display is a multivalent display technique, and the avidity of multivalent peptides overexpressed on the surface of the phage might be high. In other words, the selected single peptide after bio-panning has great potential to be further explored to be matured. No research to date has examined the mature strategy toward phage-displayed peptides.

**Scheme 1:**
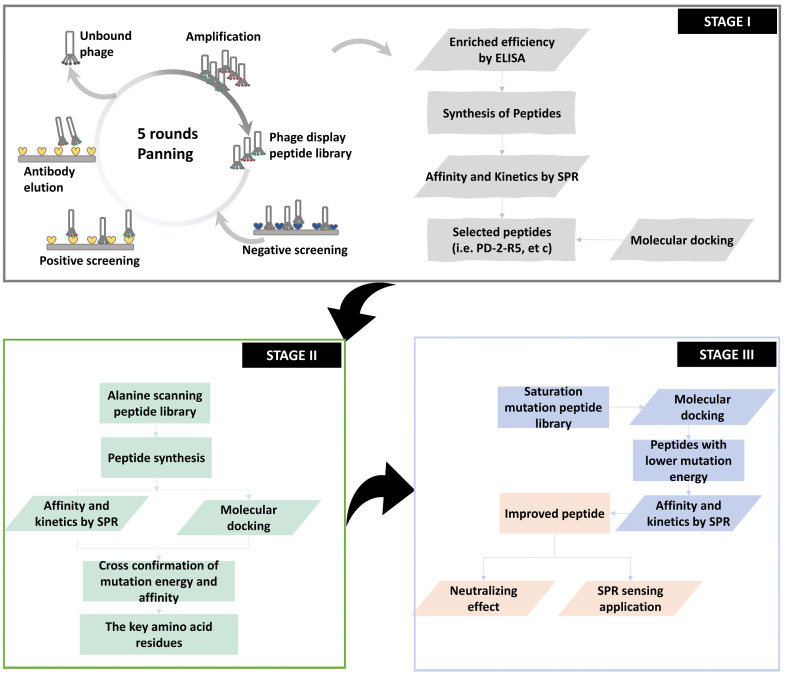
Schematic of workflow for screening peptides by phage display.

Here, we report a new and comprehensive strategy to accelerate the process of affinity-mature under the guidance of in silico prediction and ex vitro evaluation. As shown in Scheme 1, three stages compose this strategy. Stage 1 is a typical phage display, and stage 2 is the first affinity-mature round based on a single-site alanine mutation. The main finding is that we proved that our in silico molecular docking prediction model could be well supported by the affinity evaluation results via SPR, in which three key sites in the sequence are determined. It aids us to proceed the final third stage, where we performed the molecular docking on the saturation-mutated sequences with a capacity of 6859 sequences (19*19*19 sequences), selected the most potential 40 sequences ordered by the lowest mutation energy, and examined the binding affinity and kinetic parameters using SPR. Finally, we evolved two peptides with an affinity of two orders of magnitude beyond primary sequences. We then tested the neutralization potential, sensing sensitivity, and selectivity to prove the applicability of those evolved peptides.

### 2.1. Stage 1: Phage Display

We employed here *E. coli* ER2738 to display 12 linear peptides. The theoretical content of this peptide library is 4 × 10^15^ pfu, and in practice, the content would be 1.5 × 10^12^ pfu. The phage display screening cycle of affinity peptides targeted ricin or ricin agglutinin (RCA120) includes (i) the “coating-elution-amplification-enrichment” procedure, (ii) the combination of positive screening of target proteins and reverse screening of non-target proteins, and (iii) the bound phages were eluted by the ricin-specific mAb MIL50 or RCA120-specific mAb 7D10. After that, one aliquot of the elute was tested for positive plaque by ELISA. After the DNA sequences were determined and translated, we obtained and aligned the peptide sequences. The other aliquot of the elute was amplified and enriched for the next round of panning. The investigated parameters include the target input, the content of Tween-20 in the elute, positive and reverse screening, and the addition of antibodies to the elution solution, so as to achieve specific peptide panning. A total of five pannings were completed.

**Figure 1:**
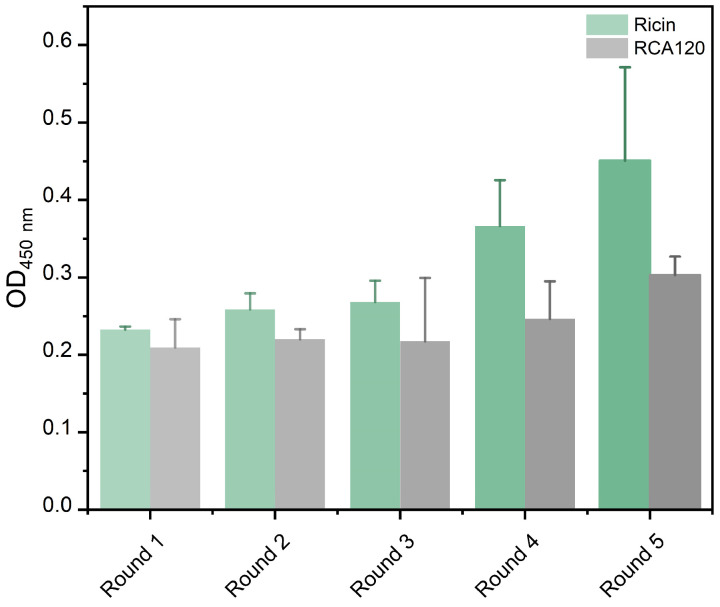
The OD_450nm_ value of an enriched peptide library against ricin or RCA120 in each round.

The recovery efficiency of phages was measured by the equation of “recovery = output phage/input phage”. As shown in Table 1, the results indicated that specific phages were effectively enriched in every cycle. At the same time, the accumulation of specific phages was confirmed by ELISA following five rounds of panning, and the OD_450nm_ value of specific phages to ricin or RCA120 gradually increased (Figure 1).

#### 2.1.1. ELISA Evaluation of Enriched Sequences

**Figure 2:**
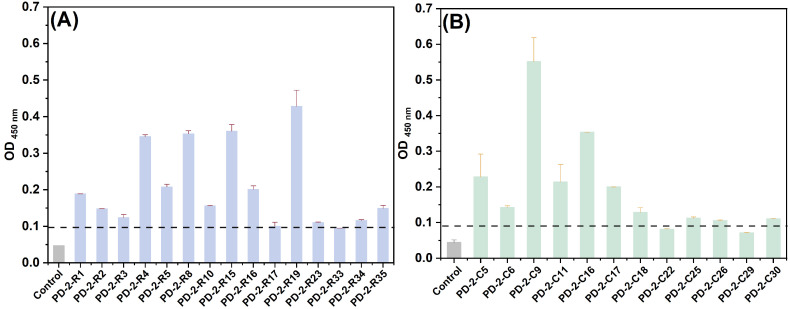
The binding OD_450nm_ of monoclonal phages evolved from the fifth pool for ricin (**A**) and RCA120 (**B**) by ELISA. The dotted line was a positive boundary line.

Subsequently, we evaluated the affinity efficiency of enriched phage clones with target toxins by ELISA towards the monoclonal phages isolated from the fifth panning pool. Twenty-one phage clones for ricin and seventeen phage clones for RCA120 were picked up from the Luria–Bertani medium isopropyl β-D-thiogalactoside 5-bromo-4-chloro-3-indolyl β-D-galactopyranoside (LB-IPTG-X-gal) plates and used to infect *E. coli* ER2738 for phage amplification and isolation, respectively. Regarding the DNA sequences and amino acid sequences of all plaques, 15 peptide sequences from 21 phage clones for ricin and 12 peptide sequences from 17 phage clones for RCA120 were displayed. We set the original phage library as a negative control, and if the ratio of the OD_450nm_ of the sample to OD_450nm_ of the negative control was greater than 2, the presented phage clone was regarded as a positive clone. As shown in Figure 2, 14 of 15 phages for ricin and 10 of 12 phages for RCA120 were found to be capable of binding to the input target biotoxin. Among them, for ricin, PD-2-R23 had a higher frequency from six repetition phage clones. PD-2-R2, PD-2-R5, and PD-2-R33 also appeared two times. Similarly, for RCA120, PD-2-C5 had a higher frequency from five repetition phage clones, and PD-2-C9 appeared two times.

#### 2.1.2. SPR Evaluation of Enriched Sequences

**Figure 3:**
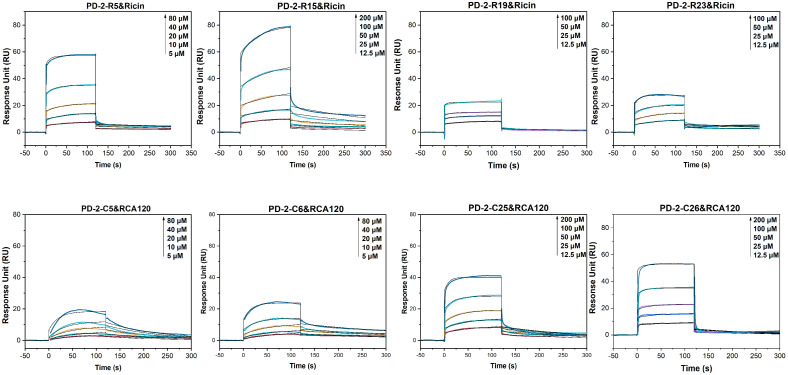
The multi-cycle kinetics (MCK) of peptides towards ricin or RCA120 using SPR.

We investigated the affinity and kinetics parameters of selected peptides toward the target toxin by a multi-cycle kinetics (MCK) approach (Figure 3 and Figure S1, Table 2 and Table S1). All curves show the characteristics of moderate association and moderate dissociation rates, indicating a moderate binding affinity.

According to the association and dissociation curves and K_D_ value of the peptides towards ricin, candidate peptides with higher affinity were further screened. For ricin, as shown in Table 2, the K_D_ values of three peptides to ricin ranged from 2 μM to 4 μM, and PD-2-R15 has the lowest binding affinity with a K_D_ of 35 μM. For RCA120, the K_D_ values of four enriched peptides to RCA120 were 4 μM, 9.6 μM, 16 μM, and 45 μM.

#### 2.1.3. Molecular Docking of Enriched Peptides and Ricin

Molecular docking is an essential tool to reveal the interaction and binding sites of peptides and target molecules. It lays the foundation for better understanding and precisely predicting the interaction modes for various kinds of molecules. The essential software includes Discovery Studio, H-docking, AutoDock, Schrödinger, etc. Here, we selected the online H-docking software to simulate the according interactions. H-DOCK is an online web server for protein–protein and protein–DNA/RNA docking, which was a hybrid docking algorithm of template modeling and free docking to improve the success rate of prediction [25]. It has advantages, including specifically setting the residues of binding sites to limit docking range, supporting amino acids sequence input, docking scores, and root mean square deviation values [26].

Based on our previous work on the molecular docking between ricin and oligonucleotides substrates [27,28], in which 13 residues composing primary and secondary pockets of ricin were chosen, and the fact that the docking results meet the experimental well, as a result of this, the same docking parameters applied to the interaction between peptide and ricin. In the H-dock, the fix of the primary and secondary pockets of ricin is reasonable because we also performed a total “blind” docking of the peptide to ricin in Discovery Studio without setting receptor binding site residues. The relative docking sites were similar, indicating that the peptides are most inclined to be bound in the active pockets of ricin and that our H-dock model was reliable.

**Figure 4:**
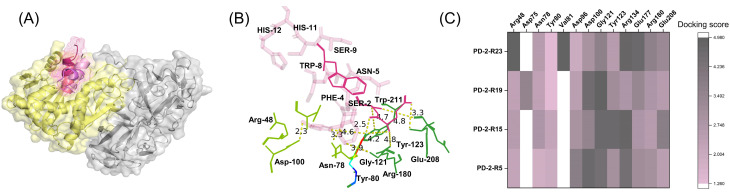
Molecular docking of peptides with ricin (**A**), in which yellow: ricin A chain; grey: ricin B chain; red: PD-2-R5; cyan: PD-2-R15; green: PD-2-R19; and pink: PD-2-R23; the interaction sites (**B**) in which pink: PD-2-R5; forest green: primary active pocket of ricin, and split pea green: second active pocket of ricin; and the heat map of site interactions between four peptides and ricin (**C**).

As shown in Figure 4, PD-2-R5, PD-2-R15, PD-2-R19, and PD-2-R23 interacted with the active pocket of ricin. For PD-2-R5, it mainly interacted with critical amino acid residues in the active pocket of ricin through hydrogen bonds of the primary pocket of ricin, in which TRP8 in that peptide interacted with Tyr80 and Asn78 of the “entrance” of the secondary pocket of ricin [29] through hydrogen bonds, respectively. GLN1 interacted with Tyr80, Val81, Gly121, Tyr123, Glu177, Arg180, and Trp211 of the primary pocket, and SER2 interacted with Tyr123 and Arg180 of the primary pocket through hydrogen bonding. The distances between the interacting amino acid residues were all within 5 Å, and the ZRank of the peptide PD-2-R5 and ricin was −169.31. It was suggested that PD-2-R5 interacted with ricin in a stable posture. Other peptides have similar interaction ways in which multiple interaction forces such as hydrogen bonds, Pi-alkyl interactions, and other kind interaction forces. For a detailed discussion, refer to Supplementary Materials.

For PD-2-R5, the panning frequency was two, the affinity frequency of OD_sample (450nm)/control (450nm)_ was 4.39, the K_D_ was 4.7 μM, and it could interact with the primary and secondary pocket of ricin through hydrogen bonds compared with other peptides. PD-2-R5 interacted more with the secondary pocket residues of ricin (Figure 4C). To sum up, PD-2-R5 was chosen as the further peptide in the following stages.

### 2.2. Stage 2: In Silico–Ex Vitro Cross-Confirmation on PD-2-R5-Derived Alanine Scanning Peptide Library

The most convenient way to construct a limited single-site peptide library is using an alanine (ALA) scanning way in which alanine is the simplest structural amino acid instead of according to residues [30]. As shown in Table 3, alanine mutants of PD-2-R5 peptides are sorted from low to high mutation energy. According to the in silico molecular docking results, which were calculated from the Calculate Mutation Energy (Binding) module of Discovery Studio in a semi-flexible mode, mutation energy is neutral between −0.5 and 0.5 kcal/mol, which means that the mutation does not affect affinity; mutation energy greater than 0.5 kcal/mol implies that the mutation causes a decreased affinity and a weakened interaction, i.e., having a destabilizing effect. Mutational energy less than −0.5 kcal/mol indicates a stabilizing effect, in which the mutation results in an increased affinity and enhanced interaction. The results from the changes in mutation energy suggest that the key amino acid residues of the interactions between the peptide and ricin are GLN1, PHE4, ASN5, TRP8, and ASN11, according to mutation energy values 2.46, 0.57, 0.81, 2.8, and 0.96 kcal/mol.

Meanwhile, we determined the affinity and kinetic parameters of PD-2-R5 peptide-derived ALA scanning mutants with ricin using SPR (Figure S2 and Table S2). The results showed that most of the K_D_ values of this mutation peptide library and ricin were increased. Among them, the K_D_ values derived from the TYR3 > ALA, PHE4 > ALA, ASN5 > ALA, TRP8 > ALA, and SER9 > ALA, ASN11 > ALA peptides were much larger to be determined. It is speculated that these amino acid sites might be the key amino acid sites for the interaction between PD-2-R5 peptide and ricin. The K_D_ values of CYS10 > ALA were about 10 times higher than the original K_D_ values, which might be the second key amino acid sites.

Combining the results from in silico mutation energy and ex vitro SPR-measured binding affinity values, the amino acid residues of PHE4, TRP8, and ASN11 were determined to be the most dominant residues that contributed to the binding. Hereafter, we selected and submitted PHE4, TRP8, and ASN11 for the next stage.

### 2.3. Stage 3: In Silico Predication on PD-2-R5-Derived Satuation Mutation Peptide Library Guided the SPR Evaluation

Considering the library capacity and the computility, we construct an in silico saturation mutation peptide library derived from PHE4, TRP8, and ASN11 in the PD-2-R5 peptide sequence, comprising 6859 mutants. The first 40 mutants with a sorted mutation energy from low to high are listed in Table 4. The mutation energy is between −4.28 kcal/mol and −1.95 kcal/mol, indicating that this exhaustive method could reasonably provide potential peptides with better binding affinity.

**Figure 5:**
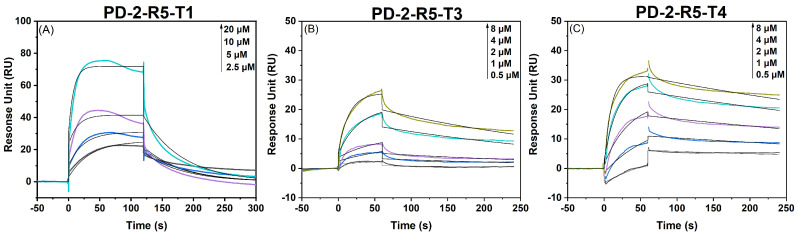
The multi-cycle kinetics of saturation-mutated peptides PD-2-R5-T3 and PD-2-R5-T4 and ricin using SPR.

The affinity and kinetic parameters of these 40 saturated mutants for ricin were determined using SPR. The results showed that the K_D_ values of 40 peptides were between 0.23 μM and 15.6 μM, where the peptide PD-2-R5-T1 (PHE-4 > ARG, TRP8 > TYR, and ASN11 > TRP) had a K_D_ of 1.35 μM for binding to ricin (k_a_ = 8.64 × 10^3^ M^−1^ s^−1^; k_d_ = 1.00 × 10^−2^ s^−1^). Compared with the original PD-2-R5 sequence (K_D_ = 4.7 μM), the affinity is increased by about 3.5 times. The PHE4 > TRP, TRP8 > PHE, and ASN11 > ARG mutated peptide (named PD-2-R5-T3) had a K_D_ of 230 nM for binding to ricin (k_a_ = 4.32 × 10^3^ M^−1^ s^−1^; k_d_ = 1.00 × 10^−3^ s^−1^); the affinity is increased by about 20 times. The peptide, named PD-2-R5-T4, formed from PHE4 > ARG, TRP8 > PHE, and ASN11 > ARG, had a K_D_ of 900 nM for binding to ricin (k_a_ = 5.68 × 10^3^ M^−1^ s^−1^; k_d_ = 5.00 × 10^−3^ s^−1^). The affinity is increased by about five times in comparison with the original PD-2-R5 sequence (Figure 5).

In particular, the kinetic curves of SPR of PD-2-R5-T3 and PD-2-R5-T4 unveil a change from the original fast dissociation rate of PD-2-R5 to a relatively slow dissociate rate, demonstrating that the binding peptides are harder to be dissociated from the target protein after association. These experimental results lay a solid foundation for this proposed affinity-mutation strategy.

At the same time, we analyzed the interaction sites of peptides with ricin by molecular docking. Peptides interacted with the active amino acids in the primary and secondary pockets of ricin (Table S3). For example, for the PD-2-R5-T3 peptide, SER2, TYR3, GLN1, and TYR3 interacted with the Arg48 and Asn78 of the secondary pocket of ricin within 4.5 Å, respectively. TYR3, CYS7, and PHE8 interacted with Tyr80 of ricin within 4 Å, and ASN5 interacted with Tyr80 of ricin at 4.92 Å. GLN1, TYR3, TRP4, ALA6, CYS7, PHE8, CYS10, and HIS12 bounded to Asp96, Asp100, Gly121, Tyr123, Glu177, Arg180, Glu208, and Trp211 of the primary pocket of ricin within 5 Å, respectively.

### 2.4. Potential Neutralizing Effect towards the Affinity-Matured Peptides

**Figure 6:**
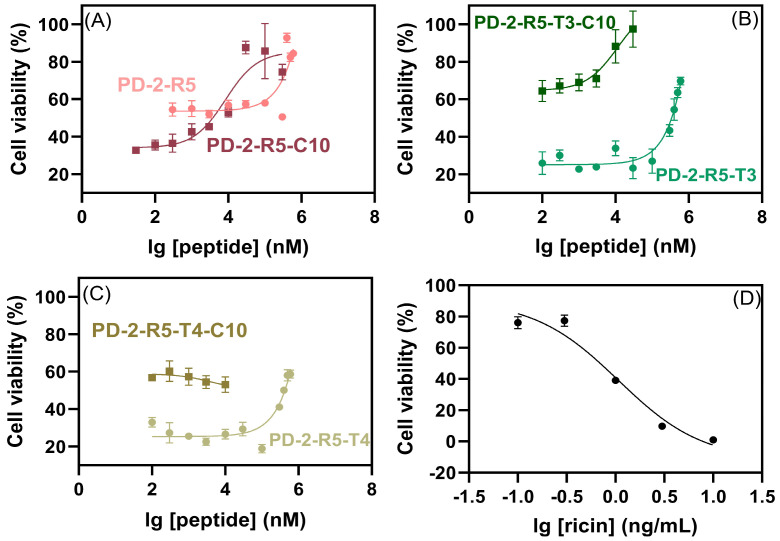
The neutralizing effect of PD-2-R5 and PD-2-R5-C10 peptides (**A**), PD-2-R5-T3 and PD-2-R5-T3-C10 peptides (**B**), PD-2-R5-T4 and PD-2-R5-T4-C10 peptides (**C**) on ricin, and the curve of cytotoxicity with ricin only ((**D**), IC_50_ = 1.1 ± 0.1 ng/mL, R^2^ = 0.965) by CCK-8 assay.

We evaluated the neutralizing effect of peptides on HeLa cells exposed to ricin using CCK−8assay (Figure 6). Peptides with different concentrations were incubated with ricin and then co-incubated with cells. The cell survival rates with ricin at IC_50_ (1 ng/mL) without peptide were 54 ± 0.7% (PD-2-R5), 31 ± 2.1% (PD-2-R5-C10), 32 ± 4.9% (PD-2-R5-T3), 59 ± 5.3% (PD-2-R5-T3-C10), 32 ± 4.5% (PD-2-R5-T4), and 56 ± 1.7% (PD-2-R5-T4-C10), as we kept all such cell survival rates at this zero point as 44 ± 14%. When the PD-2-R5, PD-2-R5-T3, and PD-2-R5-T4 peptides were up to 400 μM, the cell survival rate reached more than 60%, and the EC_50_ was 132 ± 26 μM, 340 ± 78 μM, 800 ± 50 μM, respectively. According to the results, we speculated that the peptides might not enter the cell. We then modified the peptides with a medium-chain fatty acid (C10) with a membrane-penetrating ability and measured the neutralization activity again. The results showed that the EC_50_ was 8.2 ± 0.05 μM for PD-2-R5-C10, and the cell survival was more than 60% when PD-2-R5-C10 was 30 μM, which could protect HeLa cells against ricin damage. The EC_50_ of PD-2-R5-T3-C10 was 9.2 ± 0.05 μM and the cell survival was more than 80% when PD-2-R5-C10 was 10 μM. Both peptides were comparable in their EC_50_. Even though it revealed no direct correlation between affinity and neutralizing cytotoxic activity, the largest survival rate could reach 90% with an increase of 70% contributed from PD-2-R5-C10. Considering that there are no commercial or successful antidotes against ricin, even a tiny improvement should be appreciated.

### 2.5. The Determination of Ricin Using SPR via Affinity-Mature Peptide

Through an in silico–ex vitro iterative affinity maturation strategy, we obtained PD-2-R5-T3 and PD-2-R5-T4 peptides as the matured peptides with higher affinity. We then developed an SPR measurement method for ricin based on an introduction of antifouling peptide linkage to reduce the non-specific adsorption of the peptides. In 2012, Jiang et al. reported a new antifouling peptide, the PPPP-EKEKEKE sequence [31], including a -PPPP- linkage and a -EKEKEKE- hydration layer. The -PPPP- linkage can form a rigid hydrophobic helical secondary structure and anchor to the chip surface, and the -EKEKEKE- can form an ordered self-assembled monolayer with high surface density.

Benefiting from both PD-2-R5-T3 and PD-2-R5-T4 peptides as suitable recognition modules with a feature of fast association rate and slow dissociation rate, here we designed two functional sensing peptides, whose sequences are composed of a biotinylated moiety, antifouling peptide, and PD-2-R5-T3 or PD-2-R5-T4 peptides. The conjugated time of 15 min offered 1456 RU and 1560 RU for Bio-PPPP-EKEKEKE-PD-2-R5-T3 and Bio-PPPP-EKEKEKEKE-PD-2-R5-T4 peptides.

**Figure 7:**
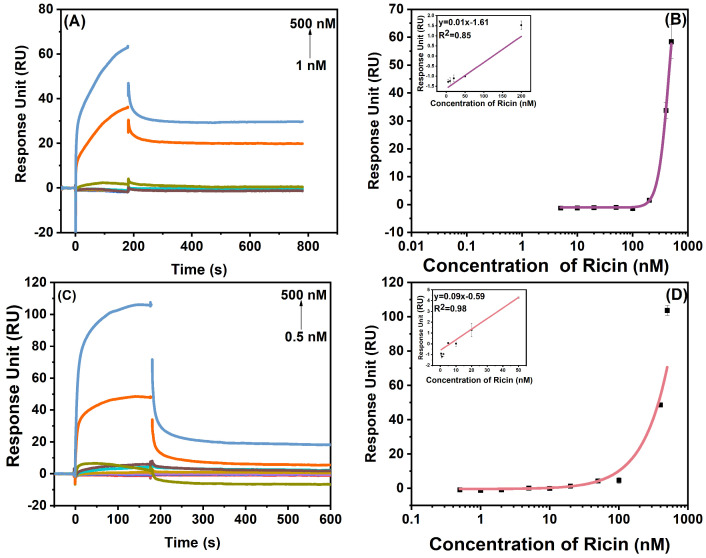
The SPR response of ricin at a concentration from 1 nM to 500 nM based on Bio-PPPP-EKEKEKE-PD-2-R5-T3 (**A**) and Bio-PPPP-EKEKEKE-PD-2-R5-T4 (**C**). The titration and linear curves (inset) of SPR quantification on Bio-PPPP-EKEKEKE-PD-2-R5-T3 (**B**) and Bio-PPPP-EKEKEKE-PD-2-R5-T4 (**D**).

As shown in Figure 7, based on the Bio-PPPP-EKEKEKE-PD-2-R5-T3 peptide, ricin could be titrated from 1 nM to 500 nM, and the linear range was from 1 nM to 200 nM with a LOD of 1 nM. Similarly, the titration range was from 0.5 nM to 500 nM. The linear range was from 0.5 nM to 50 nM based on the Bio-PPPP-EKEKEKE-PD-2-R5-T4 peptide, and the LOD was 0.5 nM.

**Figure 8:**
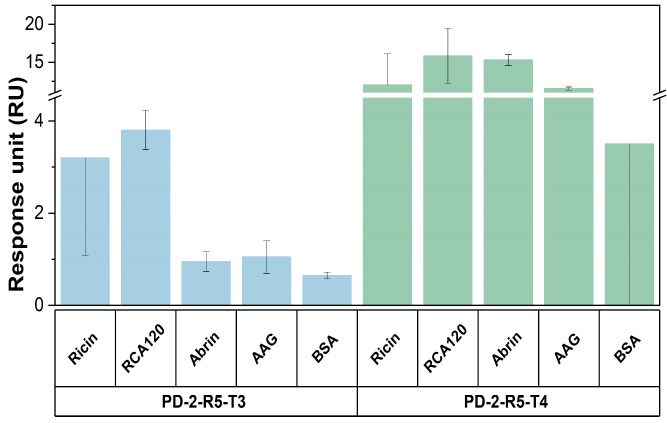
The selectivity of this SPR assay based on Bio-PPPP-EKEKEKE-PD-2-R5-T3 and Bio-PPPP-EKEKEKE-PD-2-R5-T4.

In addition, the selectivity was examined towards structural similar proteins RCA120, abrin, abrin agglutinin (AAG), and negative control bovine serum albumin (BSA) in a mediate concentration of 10 nM (Figure 8). The results show that the Bio-PPPP-EKEKEKE-PD-2-R5-T3 peptide has a good discriminative capability to abrin, AAG, and BSA but has some cross-reactivity to a structurally similar protein, RCA120. The Bio-PPPP-EKEKEKE-PD-2-R5-T4 can be regarded as a common recognition module for these four similar RIP-II proteins. Possible reasons are, first, ricin has a high structural similarity with RCA120 and abrin, all three are AB type biotoxins with a high sequence homology of 93% and 84% between the A chain and B chain of ricin and RCA120, and 60% and 40% of the A chain and B chain of ricin with abrin, the sequence homology of 69.8% between abrin and AAG, the structural and sequence similarity might lead to cross-reactivity [32,33]. Second, the peptide Bio-PPPP-EKEKEKE-PD-2-R5-T4 is a short peptide, which limits its space and site interactions with the target protein.

So far, there are several peptides and peptide derivatives binding to ricin. The 12 mer peptide inhibitor P3 with K_D_ of 1 μM was screened by the FliTrx random peptide combination library in 2003 [21], which could bind to ricin. The C4 peptide was obtained by the phage display peptide library, which could bind to the subdomain ricin B chain [22]. Peptide-conjugated pterins could competitively inhibit the active site of the ricin A chain, in which the IC_50_ values were from 6 μM to 115 μM [34]. Compared to previous counterparts, our 12 mer peptides were screened by phage display through five rounds and further matured by the new in vitro–ex vitro strategy, which has a higher affinity with lower K_D_ and considerably good EC_50_ values. For example, peptide PD-2-R5-T3 evolved with K_D_ values of 230 nM and with an EC_50_ of 11.7 μM after chemical modification by a C10 fatty acid to prompt the cell penetration. It indicates the neutralization possibility of this kind of matured peptide even at an early stage. However, we can already exert the sensing probability of such peptides; in our developed SPR method, peptide-based biosensing was established with a sensitivity of 0.5~1 nM.

## 3. Conclusions

To unveil the potential of the phage display technique, we created and examined a mature affinity strategy based on an in silico-ex vitro iteration evolution after completing the normal phage display process. We discussed the key parameters to fulfill the successful results of obtaining peptides with a higher affinity at the nM level towards biotoxin ricin, in which PD-2-R5-T3 and PD-2-R5-T4 achieved 5 and 20 times higher than the original peptide, PD-2-R5. We then tested the neutralizing activity and sensing potential of those two peptides, and the LOD of 1 nM and 0.5 nM for ricin was achieved. We hope this screening and affinity mutation strategy can provide a new concept for in vitro evolution of peptides. In further work, we will keep the focus on exerting the potential neutralization ability of matured peptides by virtue of the characteristics of ease of preparation and modification and better binding ability from the aspect of balancing the parameters of binding affinity, solubility, stability, and membrane-penetrating ability. We also expect that those developed peptides can exert their unique values on the first response to ricin in the biodefense field.

## 4. Materials and Methods

### 4.1. Materials

Ricin, RCA120, abrin, and AAG with milligram amounts were prepared in-house with an electrophoretic purity greater than 95% [27]. Peptides and biotinylated peptides were synthesized and characterized by Synpeptide Co., Ltd. (Nanjing, China). The 4-(2-hydroxyethyl) piperazine-1-ethane sulfonic acid (HEPES), BSA, Tween-20, and ethylene diamine tetraacetic acid (EDTA, 99.995% purity) were obtained from Sigma-Aldrich Co. (St. Louis, MO, USA). The 1-ethyl-3-(3-dimethyl aminopropyl)-carbodiimide hydrochloride (EDC) and N-hydroxysuccinimide (NHS), ethanolamine (1 M, pH 8.5), sodium acetate–acetic acid (CH_3_COONa- CH_3_COOH) solutions with pH 4.0, CM5 chip and SA chip were purchased from Cytiva (Danaher Corporation, Washington, DC, USA). LB, PEG 8000, IPTG, and X-gal, 3,3′,5,5′-tetramethylbenzidine (TMB) and trihydroxymethyl aminomethane (Tris) were obtained from Solarbio Science & Technology Co., Ltd. (Beijing, China). The MIL50 antibody and 7D10 antibody were donated by Prof. Feng Jiannan’s group from the Beijing Institute of Pharmacology and Toxicology, China. The human cervical cancer model cell line (HeLa) was obtained by ATCC (Shanghai, China). The RPMI-1640 medium and fetal bovine serum (FBS) were obtained from Gibco (Grand Island, NY, USA). The Ph.D.-12^TM^ phage display peptide library kit and HRP Anti-M13 antibody was purchased from New England Biolabs (Ipswich, MA, USA). The CCK-8 kit was purchased from Biyuntian Biotechnology Co. (Shanghai, China). HCl, NaCl, NaOH, NaHCO_3_, and NaHCO_3_ were obtained from Sinopharm Chemical Reagent Co. (Beijing, China). All reagents are of analytical purity or better. Ultrapure water (18 MΩ·cm) was generated by a Milli-Q A10 water purification system (Millipore, MA, USA).

**Caution:** Ricin, RCA120, abrin, and AAG are highly toxic toxins. All experiments should only be operated carefully in a well-ventilated safety hood with protective measures. All samples should be completely decontaminated by autoclaving and lye after the experiment.

### 4.2. Screening of Peptide by Phage Display

A Ph.D.-12^TM^ phage display peptide library containing 1.5 × 10^13^ pfu/mL and 2.7 × 10^9^ transformants was used to screen for targeting ricin or RCA120. Slight modification was made according to the previous literature [35,36]. Briefly, ricin (10 µg/mL in 0.1 M NaHCO_3_, pH 8.6) and RCA120 (10 µg/mL in 0.1 M NaHCO_3_, pH 8.6) were added to 96-cell plates (100 μL/well), respectively. After overnight incubation at 4 °C, the coating solution was removed, and the blocking solution (0.1 M NaHCO_3_, pH 8.6, 5 g/L BSA) was filled. After incubation at 4 °C for 2 h, the plate was rapidly washed six times with a TBST buffer (50 mM Tris-HCl, 150 mM NaCl, and 0.1% Tween-20, pH 7.5). A 4 × 10^4^ pfu/mL phage from the Ph.D.-12^TM^ phage display peptide library was added to each well and shaken for 60 min at room temperature. Unbound and weakly bound ricin or RCA120 phage was removed, and bound molecules were eluted with 0.2 M glycine-HCl (pH 2.2) and a 1 g/L BSA buffer. After gentle shaking for 10 min, the eluate was transferred to another clean microcentrifuge tube. The 1 M Tris-HCl (pH 9.1) was used to neutralize the above eluent. Eluted phage was amplified in *E. coli* ER2738. After the culture was centrifuged at 10,000 rpm for 10 min at 4 °C, the supernatant was transferred to a new centrifuge tube and re-precipitated with a 1/6 volume of PEG/NaCl (20% *w*/*v* PEG 8000, 2.5 M NaCl). After incubation on ice for 60 min, centrifuge at 4 °C at 10,000 rpm, the supernatant was removed, and the pellet was resuspended in 200 μL TBS (0.02% NaN_3_). The eluate was amplified, and stocks of phage clones were stored at 4 °C.

The titers of the amplified phage particles were tested after elution and amplification in *E. coli* ER2738. In particular, during the 2–5 rounds of screening of ricin, phages were first incubated with RCA120 for negative panning, which removed the affinity molecules binding to RCA120. Then, the unbound phage was incubated with ricin for positive screening to obtain affinity molecules bound to ricin. Finally, bound phages were competitively eluted with ricin-specific antibody MIL50 (20 ng/mL, 100 μL) and gently shaken for 10 min. The eluate was transferred to another clean microcentrifuge tube. Similarly, screening for affinity molecules targeting RCA120 in 2–5 rounds was used as a control, and phages were first incubated with ricin to remove negative molecules. Then, the unbound phages were incubated with RCA120 to obtain positive affinity molecules, and finally, the RCA120-specific antibody 7D10 (20 ng/mL, 100 μL) was applied to competitively elute the binding phages. The high-affinity phages were screened for five rounds of the above-repeated operations.

After the phage was amplified in *E. coli* ER2738 and centrifuged, 500 μL of the phage supernatant was transferred to a sterile new centrifuge tube. Then, 200 μL of PEG/NaCl was added and incubated for 10 min at room temperature. Next, the pellet was resuspended in 100 μL of an iodide buffer and 250 μL of ethanol and incubated for 10 min at room temperature after centrifugation at 10,000 rpm. Then, the pellet was washed with 70% ethanol and centrifuged. After that, the pellet was resuspended in 30 μL of a TE buffer (10 mM Tris-HCl, 1 mM EDTA, pH 7.5). The phage DNA was extracted from 5 μL of the above template solution with 5′-CCCTCATAGTTAGCGTAACG-3′ as the primer using the Sanger method.

### 4.3. ELISA

Aliquots of ricin (10 μg/mL in 0.1 M NaHCO_3_, pH 8.6) and RCA120 (10 μg/mL in 0.1 M NaHCO_3_, pH 8.6) were coated on a 96-well plate and incubated at 4 °C overnight [36,37]. The excess target molecules were removed and a blocking solution was filled, which was blocked at 4 °C for 2 h. After removing the blocking solution, the plate was washed six times with a TBST-5 (50 mM Tris-HCl, 150 mM NaCl, 0.5% Tween-20, pH 7.5) buffer. Phage clones were incubated in ricin or RCA120-coated ELISA plates at room temperature for 2 h with shaking. Then, the HRP-labeled M13 antibody was added to each well and incubated at room temperature for 1 h. After washing the plate six times with TBST-5, the TMB solution (100 μL/cell) was reacted for 30 min, and the absorbance at 450 nm was measured by a microplate reader (Tecan M1000 PRO, Tecan, Männedorf, Switzerland).

### 4.4. SPR

The binding affinity of the screened peptides to the target molecule was evaluated using SPR (Biacore T200, Cytiva, Washington, DC, USA). The SPR procedure was performed as previously described [8]. Briefly, the target molecule was first immobilized on the CM5 chip by covalent coupling. Ricin (50 μg/mL, dissolved in pH 4.0 NaAc-HAc solution) and RCA120 (50 μg/mL, dissolved in pH 4.0 NaAc-HAc solution) were activated by EDC/NHS with their carboxyl groups reacted with dextran amino groups on the chip. The chip was then blocked with ethanolamine. Peptides of different concentrations (dissolved in 0.01 M HEPES, 0.15 M NaCl, 0.05% Tween 20, pH 7.4) were injected into the flow cell (Fc) on the chip surface. The flow rate was 30 μL/min, the injection time was 120 s, the dissociation time was 180 s, and the regeneration was performed with 50 mM HCl for 30 s to evaluate the affinity and kinetic parameters. The Fc1 channel was used as the reference channel without any protein covalent modification. The baseline was established by injecting HEPES into the sensor surface. Finally, the experimental data were recorded and analyzed using Biacore control software 3.0. The change in the response value was calculated by subtracting the signals from the sensing and control channels.

The multiple-curve kinetics mode was used to determine the binding affinity and kinetic parameters for peptide and target protein, and the sensorgram curves were analyzed by a kinetics model of 1:1 binding. In this model, ka represents the association rate constant, kd represents the dissociation rate constant, and K_D_ is the dissociation equilibrium constant, indicating the degree of dissociation in equilibrium, and the lower the K_D_, the stronger the affinity.

### 4.5. Molecular Docking

The interaction of peptides with ricin or RCA120 was simulated by Discovery Studio 2021 Client (DS) software and H-DOCK (http://hdock.phys.hust.edu.cn/, accessed on 23 December 2022). In the software, the binding of peptides to target molecules was simulated in a semi-flexible manner. Molecular docking was carried out as previously described [28]. Briefly, the peptide sequence was folded by PEP-FOLD_3_ (https://mobyle.rpbs.univ-paris-diderot.fr/cgi-bin/portal.py#forms::PEP-FOLD, accessed on 1 December 2022) and optimized to form a three-dimensional structure. At the same time, the three-dimensional structures and sequences of ricin (PDB ID: 3RTJ) and RCA120 (PDB ID: 1RZO) were downloaded from the PDB protein database. All heteroatoms and other ligand molecules in the molecular structure were removed by DS. The preparation for docking was performed through the prepare protein module, and then the interaction site analysis between the peptide and the target protein was performed through the analyze protein module in DS software (“blind” docking) or the H-DOCK (specifying the interaction sites). All peptides towards ricin were first analyzed using DS software without specifying any residues of binding sites, in which the binding poses of peptides and proteins were evaluated in the ZDOCK of the Dock proteins module. The binding poses were scored by ZRank. The lower the ZRank value, the more stable the steric binding. The detailed interaction sites of peptides and proteins were analyzed by RDOCK of the Dock proteins module. While the interaction of peptides and proteins was analyzed by H-DOCK, the residues of binding sites of ricin were specified as including its first pocket and secondary pocket in its N-glycosylase active sites. Finally, PyMOL software (version 2.2.0, Delano Scientific LLC, Palo Alto, CA, USA) was used to carry out the simulation drawing of molecular docking results.

### 4.6. Iterative Affinity Maturation of Peptide In Silico–In Vitro

ALA mutants were constructed by replacing non-alanine residues in peptides [38,39], and the mutants were synthesized by solid-phase synthesis (Synpeptide Co., Ltd., Nanjing, China). The binding affinity and kinetic parameters of alanine mutants to target proteins were evaluated using SPR. A higher-specificity peptide PD-2-R5 was obtained, and further experimental research was carried out.

After amino acid mutation to ALA, the key amino acid residues in the peptide sequence were deduced. The binding mutation energy values of peptide alanine scanning mutants and ricin were calculated by the Calculate Mutation Energy (Binding) function in the Design Protein module of DS software.

After determining the key amino acids interacting with the target molecule, the saturation mutation on the peptide sequences was performed [40]. The PD-2-R5 peptide was used for saturation mutation analysis. The saturated mutations were constructed by replacing the 1–12 amino acids of the PD-2-R5 peptide one by one with 19 amino acids, except for the 4, 8, and 11 residues. The binding mutation energy values were calculated using the Calculate Mutation Energy (Binding) function in the Design Protein module of DS software between peptides and ricin. The first 40 peptides with lower mutational energy were synthesized and evaluated using SPR. All three-dimensional structures and sequences of peptide and target protein were obtained as described in the previous molecular docking.

### 4.7. CCK-8 Experiments

The neutralizing effect of the selected peptides on the toxicity caused by the target molecule was examined using the CCK-8 assay [41,42]. HeLa cells in the logarithmic growth phase were trypsinized. The supernatant was discarded by centrifugation, and the medium was added to resuspend. Next, the cell concentration was adjusted to 1 × 10^4^ cells/mL well. The cell suspension was added to a 96-well culture plate (100 μL/well) and then incubated overnight in a 37 °C and 5% CO_2_ incubator. Different concentrations of peptides were treated with ricin at 37 °C for 2 h and then were incubated with cells for 24 h. The cell culture medium was discarded, 100 μL of CCK-8 was added to each well and incubated for another 1 h. Finally, the absorbance at 450 nm was measured by a microplate reader. The concentration of ricin was 1 ng/mL.

### 4.8. The Detection of Ricin Using SPR

The peptides of bio-PPPP-EKEKEKE-PD-2-R5-T3 and bio-PPPP-EKEKEKE-PD-2-R5-T4 at 50 nM were fixed to the SA chip with a flow rate of 10 μL/min and a flow-through time of 15 min, respectively [8]. A series of ricin with different concentrations were prepared with a flow rate of 10 μL/min, injecting time of 180 s, dissociation time of 180 s, and regeneration of 1.5 mM NaOH for 30 s. All data were recorded via Biacore control software 3.0. Meanwhile, different concentrations of RCA120, abrin, AAG, and BSA were configured to examine the selectivity of this sensing method. All buffers used were 0.01 M HEPES, 0.15 M NaCl, 0.05% Tween-20, and pH 7.4.

## Figures and Tables

**Table 1:** Phage display for screening peptides.

Target Protein	Rounds	Target Input (μg)	Tween-20	Eluent	Phage Input (pfu)	Phage Output (pfu)	Recovery Efficiency
Ricin	1st round	100	0.10%	Gly-HCl (pH 2.2)	1.5 × 10^11^	8.9 × 10^4^	5.9 × 10^−7^
	2nd round	50	0.20%	MIL50 ^a^	1.5 × 10^11^	1.6 × 10^4^	1.1 × 10^−7^
	3rd round	25	0.50%	MIL50	1.5 × 10^11^	4.0 × 10^4^	2.7 × 10^−7^
	4th round	12.5	0.50%	MIL50	1.5 × 10^11^	8.0 × 10^5^	5.3 × 10^−6^
	5th round	6.25	0.50%	MIL50	1.5 × 10^11^	9.9 × 10^5^	6.6 × 10^−6^
RCA120	1st round	100	0.10%	Gly-HCl	1.5 × 10^11^	3.1 × 10^4^	2.0 × 10^−7^
	2nd round	50	0.20%	7D10 ^b^	1.5 × 10^11^	1.8 × 10^4^	1.2 × 10^−7^
	3rd round	25	0.50%	7D10	1.5 × 10^11^	7.0 × 10^4^	4.7 × 10^−7^
	4th round	12.5	0.50%	7D10	1.5 × 10^11^	7.5 × 10^5^	5.0 × 10^−6^
	5th round	6.25	0.50%	7D10	1.5 × 10^11^	9.7 × 10^5^	6.5 × 10^−6^

^a^ The concentration of MIL50 was 20 ng/mL, 100 μL. ^b^ The concentration of 7D10 was 20 ng/mL, 100 μL.

**Table 2:** The affinity of peptides to ricin and RCA120 using SPR.

	Peptide	k_a_ (M^−1^ s^−1^)	k_d_ (s^−1^)	K_D_ (μM)	Chi^2^	U-Value
Ricin	PD-2-R5	4.7 × 10^2^	2.2 × 10^−3^	4.7	6.5	0.22
	PD-2-R15	0.94 × 10^2^	3.2 × 10^−3^	35	0.79	3
	PD-2-R19	1.6 × 10^3^	3.6 × 10^−3^	2.3	0.26	12
	PD-2-R23	6.6 × 10^2^	1.5 × 10^−3^	2.4	0.24	7
RCA120	PD-2-C5	5.9 × 10^2^	9 × 10^−3^	16	0.51	3
	PD-2-C6	7.7 × 10^2^	3 × 10^−3^	4.0	0.14	2
	PD-2-C25	1.3 × 10^3^	1.6 × 10^−3^	9.6	073	4
	PD-2-C26	6.2 × 10^3^	6.3 × 10^−3^	10.3	2.43	20

**Table 3:** The sequence and the mutation energy of alanine scanning mutants peptide library.

Name	Sequence	Mutation Energy (kcal/mol)	Effect	K_D_ (μM)
PD-2-R5	QSYFNACWSCNH	-	-	4.7
PD-2-R5-1	**A**SYFNACWSCNH	2.46	Destabilizing	6.7
PD-2-R5-2	Q**A**YFNACWSCNH	−1.06	Stabilizing	4.5
PD-2-R5-3	QS**A**FNACWSCNH	−0.11	Neutral	N/A
PD-2-R5-4	QSY**A**NACWSCNH	0.57	Destabilizing	N/A
PD-2-R5-5	QSYF**A**ACWSCNH	0.81	Destabilizing	N/A
PD-2-R5-7	QSYFNA**A**WSCNH	−0.11	Neutral	3.2
PD-2-R5-8	QSYFNAC**A**SCNH	2.8	Destabilizing	N/A
PD-2-R5-9	QSYFNACW**A**CNH	−0.96	Stabilizing	N/A
PD-2-R5-10	QSYFNACWS**A**NH	−0.12	Neutral	41
PD-2-R5-11	QSYFNACWSC**A**H	0.96	Destabilizing	N/A
PD-2-R5-12	QSYFNACWSCN**A**	0.09	Neutral	3.9

N/A: not available.

**Table 4:** The top 40 saturated amino acid mutants.

No.	Sequence	Mutation Energy (kcal/mol)	pI	Solubility
T1	QSY**R**NAC**Y**SC**W**H	−4.28	8.3	−0.5
T2	QSY**W**NAC**Y**SC**W**H	−4.28	7	−1.1
T3	QSY**W**NAC**F**SC**R**H	−4.24	8.3	−0.6
T4	QSY**R**NAC**F**SC**R**H	−4.21	9.1	0
T5	QSY**W**NAC**F**SC**W**H	−3.96	7	−1.1
T6	QSY**W**NAC**F**SC**I**H	−3.72	7	−1
T7	QSY**W**NAC**Y**SC**L**H	−3.65	7	−0.9
T8	QSY**R**NAC**H**SC**R**H	−3.57	9.1	0.1
T9	QSY**W**NAC**F**SC**Y**H	−3.55	7	−1
T10	QSY**K**NAC**Y**SC**W**H	−3.4	8.3	−0.5
T11	QSY**R**NAC**F**SC**K**H	−3.37	9.1	0
T12	QSY**R**NAC**Y**SC**L**H	−3.36	8.3	−0.4
T13	QSY**R**NAC**N**SC**R**H	−3.35	9.1	0.2
T14	QSY**W**NAC**Y**SC**Y**H	−3.35	7	−1
T15	QSY**W**NAC**F**SC**L**H	−3.32	7	−1
T16	QSY**W**NAC**Y**SC**M**H	−3.24	7	−0.9
T17	QSY**W**NAC**F**SC**M**H	−3.18	7	−0.9
T18	QSY**R**NAC**F**SC**L**H	−3.13	8.3	−0.4
T19	QSY**W**NAC**F**SC**N**H	−3	7	−0.8
T20	QSY**K**NAC**F**SC**W**H	−3	8.3	−0.6
T21	QSY**R**NAC**R**SC**Y**H	−2.96	9	0
T22	QSY**R**NAC**Y**SC**Y**H	−2.91	8.3	−0.4
T23	QSY**R**NAC**Y**SC**V**H	−2.91	8.3	−0.4
T24	QSY**W**NAC**F**SC**V**H	−2.88	7	−0.9
T25	QSY**R**NAC**Y**SC**H**H	−2.84	8.3	−0.3
T26	QSY**W**NAC**F**SC**P**H	−2.83	7	−0.8
T27	QSY**R**NAC**F**SC**M**H	−2.82	8.3	−0.4
T28	QSY**R**NAC**F**SC**Y**H	−2.78	8.3	−0.5
T29	QSY**W**NAC**F**SC**H**H	−2.74	7.2	−0.8
T30	QSY**W**NAC**Y**SC**P**H	−2.71	7	−0.8
T31	QSY**R**NAC**Y**SC**N**H	−2.69	8.3	−0.2
T32	QSY**R**NAC**F**SC**N**H	−2.65	8.3	−0.3
T33	QSY**W**NAC**Y**SC**H**H	−2.63	7.2	−0.8
T34	QSY**R**NAC**F**SC**V**H	−2.6	8.3	−0.4
T35	QS**Y**RNAC**Y**SC**T**H	−2.5	8.3	−0.3
T36	QSY**W**NAC**F**SC**T**H	−2.33	7	−0.8
T37	QSY**R**NAC**F**SC**H**H	−2.32	8.3	−0.3
T38	QSY**W**NAC**Y**SC**V**H	−2.21	7	−0.9
T39	QSY**W**NAC**H**SC**V**H	−2.1	7.2	−0.8
T40	QSY**K**NAC**Y**SC**H**H	−1.95	8.3	−0.3

## Data Availability

Data are contained within the article or Supplementary Material.

## Supplementary Materials

The following supporting information can be downloaded at: https://www.mdpi.com/article/10.3390/toxins15080490/s1. Figure S1: The MCK of peptides and abrin using SPR; Figure S2: The MCK curves and kinetic fitting of alanine scanning mutants binding to ricin; Table S1: The affinity and kinetic parameters of peptides and abrin by SPR; Table S2: The affinity and kinetic parameters of alanine scanning mutants to ricin; Table S3: The interaction sites of PD-2-R5-T3 and PD-2-R5-T4 (site marked by all-capitalized three-letter symbol) with ricin (site marked by first-word capitalized three-letter symbol).
