# Supplementary material for: In Silico–Ex Vitro Iteration Strategy for Affinity Maturation of Anti-Ricin Peptides and the SPR Biosensing Application
Source: Toxins (Basel). 2023 Aug 3;15(8):490. doi: 10.3390/toxins15080490 (PMC10467137; doi:10.3390/toxins15080490)
Supplement: Supplementary file 1 [file toxins-15-00490-s001.zip › toxins-2508492-supplementary.pdf]

Supplementary Materials: In Silico–Ex Vitro Iteration Strategy for Affinity Maturation of Anti-Ricin Peptides and the SPR Biosensing Application

Zhifang Yang, Chuang Wang, Jia Liu, Lan Xiao, Lei Guo1 and Jianwei Xie

1. Molecular Docking of Enriched Peptides and Ricin

PD-2-R15 mainly interacted with key amino acids in the primary pocket of ricin through hydrogen bonds. LEU9, SER12, and LEU10 created hydrogen bonds with Tyr123 and Arg180, respectively. LEU9 and LEU10 interacted with Tyr80 and Trp211 by Pi-Alkyl at 5.43 Å and 4.79 Å.

The molecular docking results showed that the interaction of PD-2-R19 and ricin was through hydrogen bonds. Mainly, TYR3 interacted with Arg48 and Asn78 in the "entrance" of the secondary pocket. Among them, the LEU6 of PD-2-R19 interacted with Tyr80 through hydrogen bonding. ALA9, GLU12, and SER11 interacted with Tyr123 through hydrogen bonding. ALA10 and SER11 interacted with the Arg180 of ricin via hydrogen bonding. The active amino acid distance between PD-2-R19 and the ricin active pocket was within 5Å. The ZRank of the peptide and ricin was -40.09. It was shown that PD-2-R19 interacted with ricin in a stable posture and had a higher affinity.

In addition, molecular simulations of PD-R23 with ricin used H-DOCK instead of DS. The result of the complexes revealed that PRO6, SER7, ILE 8, MET10, GLY4, LEU11, ALA12, and LEU11 bound to the Tyr80, Asp96, Asp100, Gly121, Asn122, Tyr123, Glu177, Arg180, and Trp211of the primary pocket of ricin within 5 Å. THR5, PRO 6, and SER7 interacted with Asn78 in the "entrance" of the secondary pocket at 4.75 Å, 2.33 Å, and 2.75 Å, respectively.

Table S1. The affinity and kinetic parameters of peptides and abrin by SPR.

Peptide	k_a ($M^{-1}s^{-1}$)	k_d (s^{-1})	K_D (μM)	Chi^2	U-value
PD-2-R5	400.5	1.0×10^{-2}	27.8	8.18	20
PD-2-R15	99.07	1.0×10^{-2}	48.6	0.44	3
PD-2-R19	361.6	4.0×10^{-2}	138.1	0.63	9

Table S2. The affinity and kinetic parameters of alanine scanning mutants to ricin.

Peptide	k_a ($M^{-1}s^{-1}$)	k_d (s^{-1})	K_D (μM)	Chi^2	U-value
PD-2-R5	4.7×10^{2}	2.0×10^{-3}	4.7	6.5	0.22
PD-2-R5-1	6.3×10^{2}	4.2×10^{-3}	6.7	5.0	3
PD-2-R5-2	5.1×10^{2}	2.3×10^{-3}	4.5	2.3	4
PD-2-R5-3	-	-	N/A	-	-
PD-2-R5-4	-	-	N/A	-	-
PD-2-R5-5	-	-	N/A	-	-
PD-2-R5-7	2.1×10^{3}	6.8×10^{-3}	3.2	16	12
PD-2-R5-8	-	-	N/A	-	-
PD-2-R5-9	-	-	N/A	-	-
PD-2-R5-10	4.5×10^{3}	0.18	41	9	7
PD-2-R5-11	-	-	N/A	-	-
PD-2-R5-12	8.2×10^{2}	3.1×10^{-3}	3.9	3.5	2

Table S3. The interaction sites of PD-2-R5-T3 and PD-2-R5-T4 (site marked by all-capitalized three-letter symbol) with ricin (site marked by first-word capitalized three-letter symbol)

Peptide	Docking Score	Interaction sites		Distance (Å)
PD-2-R5-T3	-200.9	SER2	Arg48	3.68
		TYR3	Arg48	4.10
		GLN1	Asn78	4.32
		TYR3	Tyr78	2.49
		TYR3	Tyr80	3.46
		TRP4	Tyr80	2.39
		ASN5	Tyr80	4.92
		CYS7	Tyr80	3.05
		PHE8	Tyr80	3.03
		TYR3	Asp96	3.00
		ALA6	Asp96	3.05
		CYS7	Asp96	2.95
		CYS10	Asp96	3.89
		HIS12	Asp96	3.79
		TYR3	Asp100	2.11
		CYS7	Gly121	4.28
		HIS12	Gly121	4.67

		PHE8	Tyr123	3.26
		TRP4	Glu177	4.81
		TRP4	Arg180	2.77
		TRP4	Glu208	3.69
		GLN1	Trp211	3.52
		TRP4	Trp211	3.54
PD-2-R5-T4	-187.9	ARG11	Arg48	4.83
		ARG11	Asn78	3.49
		CYS 7	Tyr80	2.87
		PHE8	Tyr80	2.61
		SER9	Tyr80	4.29
		CYS10	Tyr80	3.39
		HIS 12	Tyr80	3.71
		ARG4	Asp96	3.79
		ASN5	Asp96	2.08
		ALA6	Asp96	4.24
		PHE8	Asp96	2.27
		SER9	Asp96	1.93
		PHE8	Asp100	3.45
		PHE8	Gly121	3.70
		HIS12	Tyr123	4.20
		HIS12	Glu177	3.68
		HIS12	Arg180	2.59
		ARG11	Glu208	4.59
		HIS12	Glu208	0.77
		ARG11	Trp211	3.41
		HIS12	Trp211	3.14

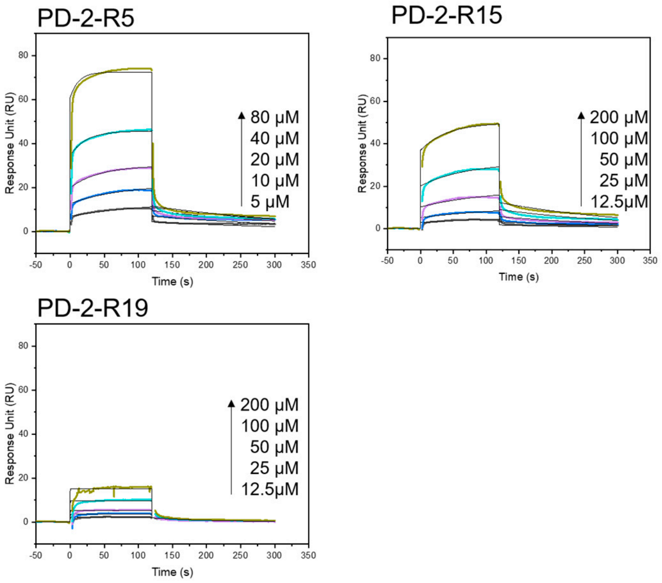

Figure S1. The MCK of peptides and abrin by SPR.

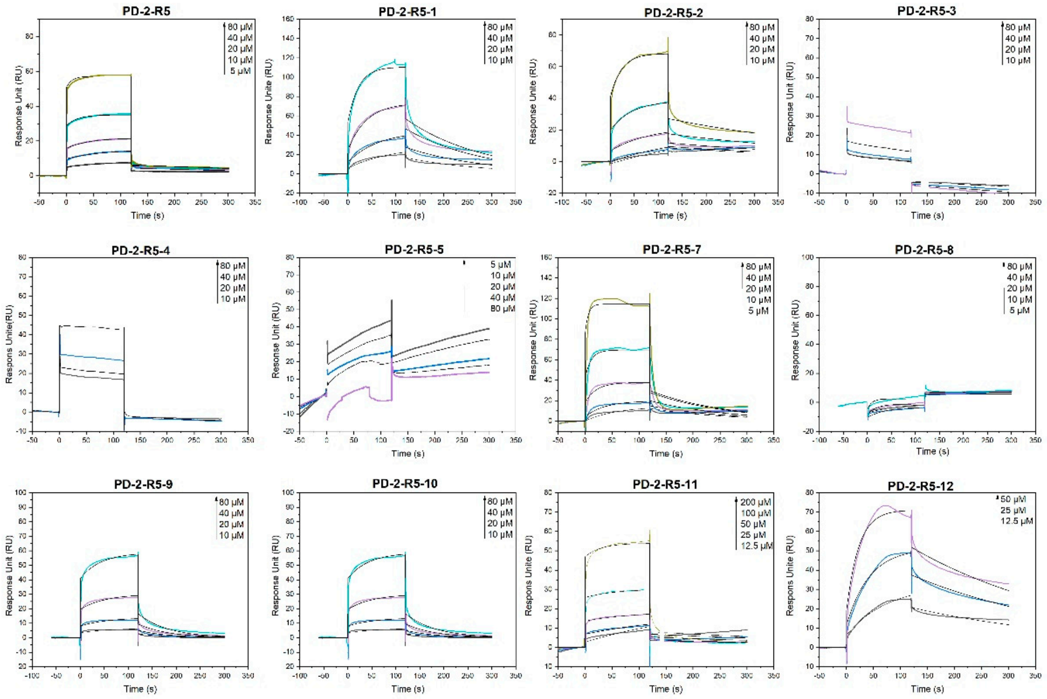

Figure S2. The MCK curves and kinetic fitting of alanine scanning mutants binding to ricin.
